# Development and Evaluation of a Single Dye Duplex Droplet Digital PCR Assay for the Rapid Detection and Quantification of *Mycobacterium tuberculosis*

**DOI:** 10.3390/microorganisms8050701

**Published:** 2020-05-10

**Authors:** Raphael Nyaruaba, Jin Xiong, Caroline Mwaliko, Nuo Wang, Belindah J. Kibii, Junping Yu, Hongping Wei

**Affiliations:** 1 Key Laboratory of Special Pathogens and Biosafety, Center for Biosafety Mega-Science, Wuhan Institute of Virology, Chinese Academy of Sciences, Wuhan 430071, China; rohuru1@gmail.com (R.N.); xiongjin@wh.iov.cn (J.X.); carolinemwaliko@gmail.com (C.M.); wangnuo@wh.iov.cn (N.W.); kibiibelindah@gmail.com (B.J.K.); yujp@wh.iov.cn (J.Y.); 2 International College, University of Chinese Academy of Sciences, Beijing 100049, China; 3 Sino-Africa Joint Research Center, Nairobi 00200, Kenya

**Keywords:** *Mycobacterium tuberculosis*, ddPCR, qPCR, Quantification, Detection, Duplex assay, Simplex assay, *IS6110*, *IS1081*

## Abstract

Droplet digital PCR (ddPCR) is a third generation of PCR that was recently developed to overcome the challenges of real-time fluorescence-based quantitative PCR (qPCR) in absolute quantification of pathogens. Few studies have been done on tuberculosis (TB) detection and quantification using ddPCR despite its many advantages over qPCR. From the few studies, none explores a single dye duplex assay for the detection and quantification of TB. In this study, steps toward developing and evaluating a duplex single dye (FAM) assay for detecting two targets (*IS6110* and *IS1081)* are clearly described using simplex and duplex experiments. To achieve this, various parameters are investigated, including annealing temperature, primer and probe concentration, sensitivity and specificity, sample concentration, and inter/intra-assay variability. From the results, primer and probe concentration, annealing temperature, and sample concentration have an effect on the position and separation of droplets in both simplex and duplex assays. The copies of target genes in a duplex assay can be estimated accurately using the threshold tool with little inter-assay (CV <1%) and intra-assay (CV <6%) variability when compared to simplex assays. The ddPCR assay specificity and sensitivity are both 100% when compared to qPCR. This work shows steps toward the detection and quantification of two targets in a single channel, enabling higher multiplexing to include more targets in future works.

## 1. Introduction

Tuberculosis (TB), a disease caused by the bacterium *Mycobacterium tuberculosis (Mtb)*, still remains the top infectious disease killer worldwide, ranking above HIV/AIDS [1]. The gold standard for TB detection and diagnosis has always been culture-based. This old fashioned gold standard technique may be effective but suffers many limitations, especially when it comes to the rapid diagnosis of TB disease [2]. Such limitations have driven scientists to come up with modernized techniques for TB diagnosis, including microscopic observation drug susceptibility (MODS) and molecular diagnostic systems [3]. Polymerase chain reaction (PCR) is a molecular diagnostic technology that is commonly used in the amplification of specific molecular targets. Biotechnological refinements of PCR led to the development of technologies that not only detect targets but also quantify them [4]. Quantification systems like the real-time quantification PCR (qPCR) and digital PCR (dPCR) were recently developed to quantify nucleic acid targets within a sample. Both these two are extensively used in the diagnosis of a variety of pathogens, including tuberculosis with dPCR, which shows many advantages over qPCR, as it does not need a standard curve during quantification [4,5].

Droplet digital PCR (ddPCR) is a third generation of PCR that was recently developed to overcome the disadvantage of dPCR during sample portioning [2]. The ddPCR method works by separating a sample into thousands to millions of droplets depending on the system, and then partitioning them to be read as either positive or negative depending on fluorescence amplitude that is exhibited. Using poison statistics, droplets that contain target sequences (positive) versus those that do not contain any targets (negative) are used to calculate the exact quantity of nucleic acid targets within a sample in copies/µL, without generating a standard curve [4]. Many methods are used in quantifying and detecting various targets using both EvaGreen and TaqMan probe assays for multiplexing in ddPCR. However, few studies exist in the application of ddPCR to detect *Mtb* [2]. In this study, two *Mtb* specific targets, *IS6110* and *IS1081*, were used for the detection and quantification of *Mtb* using ddPCR.

Since their characterization, insertion sequences (*IS*) have long been used in the diagnosis of *Mtb*. The most commonly used *IS* in tuberculosis diagnosis has always been *IS6110*. This sequence, however, is reported to be missing in some clinical samples, hence giving room for false-negative [6] and false-positive results [7]. To curb the ineffectiveness of this sequence in large parts of the world, including Southeast Asia (Vietnam) [8], another insertion sequence *IS1081* was used as a subsidiary marker in TB detection studies [9,10,11]. In our study, we develop a duplex single dye droplet digital PCR assay capable of detecting the two genes reliably in TB samples. This study also shows steps using TaqMan probe assays to perform a single dye (FAM) duplex ddPCR experiment capable of detecting and quantifying the two genes in a single channel. To the best of our knowledge, no other research has combined these two genes using a single dye based ddPCR assay in detecting *Mtb*.

## 2. Materials and Methods

### 2.1. Bacterial Strains and Ethical Considerations

Two bacterial strains *M. tuberculosis* H37Ra and *Mycobacterium bovis* bacillus Calmette-Guérin (**BCG**) were used for optimization in this study. Both the two Beijing strains were cultured and stored in our BSL 2 laboratory using solid agar (Difco™ Middlebrook 7H10 Agar) and liquid broth (Difco™ Middlebrook 7H9 Broth) medium. The conditions for reagent preparation and storage, including supplementation with OADC, were followed according manufacturer’s instructions. Another set of bacteria was provided by CDC China. Thirty samples labeled PT11HB01 to PT11HB30 and *M. tuberculosis* H37Rv were provided for testing. All the samples from CDC China were handled in our BSL 3 laboratory. No ethical permission for the study was sought as the *Mtb* samples were anonymized and no patient information could be retrieved from them.

### 2.2. Sample Processing

**Figure 1:**
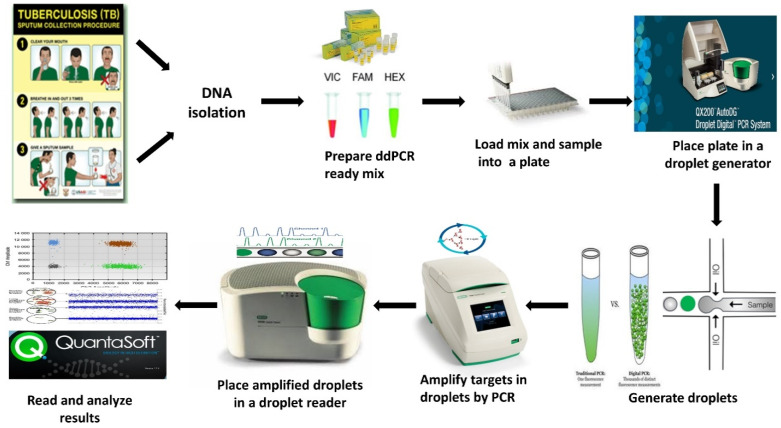
Overview of the droplet digital PCR (ddPCR) assay workflow for tuberculosis (TB) detection.

In order to study the effect of sample matrix on PCR reactions, three sputum mock samples were collected from healthy individuals and spiked with the two strains, *M. tuberculosis* H37Ra and *Mycobacterium bovis* BCG, to represent positive samples, and water used to represent controls. Spiked sputum samples and the controls were processed using the conventional N-acetyl-L-cysteine–sodium hydroxide (NALC–NaOH) method as described in the Supplementary Methods for digestion and decontamination. Following decontamination, the bacterial suspension was used for various PCR tests (qPCR and ddPCR), as shown in Figure 1.

### 2.3. DNA Extraction

To find a fast and efficient method for rapid DNA extraction involving minimal sample handling steps, a previously described method called thermocold lysis was modified [3]. In this previous method, a sample was subjected to three cycles of heating at 95 °C for 20 min and freezing at −20 °C for 20 min. In our method, we sought to optimize the method using a commercially available, automated dry water bath (0.2 mL dry bath incubator model MK-20). The DNA extraction conditions were set for three cycles of heating at 95 °C for 20 min and cooling at −7 °C for 20 min. The minimum temperature was set at −7 °C, as the dry bath incubator could achieve a minimum temperature of up to −10 °C. After extraction, all the tubes were centrifuged at 10,000× *g* at 4 °C for 10 min. The resultant supernatant was transferred to a new clean tube to be used in PCR experiments and/or stored at −20 °C until usage.

### 2.4. Assay Design and Optimization

Primers and probes specific to the two targets, *IS6110* and *IS1081*, were adopted from Chakravorty et al. [10]. The primer and probe sequences were used to generate amplicons shorter than 110 base pairs in length, as described in Table S1. All the primers and probes used in the study were synthesized by the company Sangon Biotech, Shanghai, China. In order to optimize the conditions for *Mtb* detection, different parameters including temperature gradient, primer, and probe concentration were tested to validate the assay performance. All these tests are described in detail in Supplementary Methods. 

### 2.5. Analytical Specificity and Sensitivity Tests

The specificity and exclusivity of the primers and probes for this assay were reported before on 30 different species of nontuberculous mycobacteria (NTM) and different common upper respiratory tract bacteria [10]. In our test, we tested 10 different NTM (*Mycobacterium avium, Mycobacterium marinum, Mycobacterium shimoidei, Mycobacterium kansasii, Mycobacterium asiaticum, Mycobacterium scrofulaceum, Mycobacterium gordonae, Mycobacterium chelonae, Mycobacterium fortuitum, Mycobacterium phlei*) and 5 respiratory tract bacteria (*Nocardia brasiliensis, Beijing corynebacterium, Legionella pneumophila, Bordetella pertussis*, Pneumococcus) for specificity and exclusivity. All the primers used were tested for sensitivity online first by using the basic local alignment search tool (BLAST) from the national center for biotechnology information (NCBI) [12]. After establishing the sensitivity, the assay capabilities to detect the two genes in singleplex and duplex assays were tested using laboratory-cultured *M. bovis* BCG, *Mtb*. H37Ra, and *Mtb*. H37Rv. 

### 2.6. Duplex Real-Time PCR Assay Composition

All qPCR experiments were performed in a CFX96 Touch™ real-time PCR detection system (CFX96, Bio-Rad Laboratories, Hercules, CA, USA). A 2× qPCR mixture with a final volume of 20 µL was used in all experiments. The composition of the mixture included 1.6 µL dNTP (2.5 mM for each, from Takara Biomedical Technology (Beijing) Co., Ltd, Beijing, China), 2 µL 10 × buffer (200 mM Tris-HCl pH 8.3, 200 mM KCl, 100 mM (NH_4_)2SO_4_, 20mM MgSO_4_ and 5% NONIDET P-40. SUBSTITUTE from AMRESCO Inc., Radnor, PA, USA), 0.5 µL Taq polymerase (activity unit 2.5 U/mL, expressed and purified in the laboratory), and 10.3 µL MilliQ water, 0.4 µL of 20 µM primer concentrations were added to the mixture and 1 µL of 10 µM probe was also added to the mixture. A set of two primers and probes (labeled at the 5′ end with FAM and 3′ end with BHQ 1) were used for detection. To reach a final volume of 20 µL, a sample volume of 2 µL was added. The qPCR reaction mixture was then amplified with an initial denaturation step at 95 °C for 5 s, followed by 40 cycles of denaturation at 95 °C for 5 s and an annealing (reading) step at 64 °C for 1 min. 

### 2.7. Duplex Droplet Digital PCR Assay Composition

Both *IS6110* and *IS1081* copy numbers were quantified in samples using the automated QX200™ Droplet Digital™ PCR system (Bio-Rad, Hercules, CA, USA). Briefly, before preparing the reaction mix, the sample concentration was determined using a nanodrop machine (ND-2000, Thermo Fisher, Waltham, MA, USA). Concentrations ≤60 ng/µL were considered ideal for testing. If the concentrations were higher than this, they were diluted and redetermined until the final concentration was ≤60 ng/µL. The 22 µL ddPCR mixture was composed of 11 µL of 2 × ddPCR Supermix for Probes (No dUTP), 0.7 µL of 20 µM forward and reverse primers, 0.8 µL of 10 µM probes (both labeled at the 5′ end with FAM and 3′ end with BHQ 1), DNase/RNase free MilliQ water, and 2 µL DNA sample to a final volume of 22 µL. Approximately 20,000 nanoliter-sized droplets were generated from the mixture using an automated droplet generator (QX200^TM^ AutoDG ddPCR system (Bio-Rad, Hercules, CA, USA)), as shown in Figure 1. The plate containing the resultant droplets was then heat-sealed with a pierceable aluminum foil using a PX1 PCR plate sealer (Bio-Rad, Hercules, CA, USA) set to run at 180 °C for 5 sec before being loaded into a C100 Touch^TM^ Thermal Cycler (Bio-Rad, Hercules, CA, USA) for amplification. The thermal cycling conditions with a ramp rate of 2 °C/s at every step were set to run for 10 min enzyme activation at 95 °C, followed by 40 cycles of denaturation at 94 °C for 30 s and 1 min annealing/extension at 60 °C, enzyme deactivation step set at 98 °C for 10 min, and a final hold step at 4 °C for an infinite time period. After thermal cycling, the plate containing amplified droplets was placed in a QX200^TM^ Droplet Reader (Bio-Rad, Hercules, CA, USA) for reading. Prior to reading, the QuantaSoft™ software was opened on a personal computer and wells containing the samples were labeled with the appropriate reading conditions before being run.

### 2.8. Intra- and Inter-Assay Variability and Reproducibility of Simplex and Duplex Assays

To test the intra-assay variability, six replicates of *M. bovis* BCG and *Mtb.* H37Ra were compared using simplex and duplex assays. After testing, the resultant mean and SD of the replicates data were compared to get the percentage coefficient of variability (%CV). The copies of the two genes were directly recorded for simplex assays. But for the duplex assay, further analysis using the threshold tool was conducted to estimate the exact concentration of the two target genes within the duplex assay. The threshold tool was placed slightly above the *IS1081* droplets to get the concentration of the *IS6110* gene. This was subtracted from the total concentration of the two targets after placing the threshold tool slightly above the negative droplets to get the copies of the *IS1081* gene, i.e., total copies of two targets (threshold line slightly above negative droplets) − *IS6110* copies (threshold line slightly above *IS1081* gene) = *IS1081* copies. To further test if there was a difference in copies between the simplex and duplex assays (inter-assay variability) after using the threshold tool, the mean of means of each target was compared and SD of each result obtained. This was further used to calculate the %CV between the simplex and duplex assay.

### 2.9. Data Analysis

Real-time PCR results Cq values were generated and analyzed using the Bio-Rad CFX Manager software version 2.1. For ddPCR data, QuantaSoft™ analysis software version 1.7.4 (Bio-Rad) was used to analyze the resultant droplets. Based on thresholding tools provided by the software, proportions of positive and negative droplets were discriminated by applying the threshold line above the negative droplets. For each reaction on ddPCR, the number of accepted droplets was checked, if the droplets were ≥10,000, the results were used for analysis or else discarded. Further statistical analysis was done using the GraphPad Prism Version 6.01 software.

## 3. Results

### 3.1. Analytical Specificity and Sensitivity of the Assay

The primers specificity towards various nontuberculous mycobacteria (NTM) and different bacteria were established by Chakravorty et al. [10]. An in silico test using the basic local alignment search tool (BLAST) available online from NCBI showed that the primers were highly specific to different strains of *Mycobacterium tuberculosis* complex. We also tested 15 samples including 10 NTM and five different respiratory tract bacteria that did not belong to the *Mycobacterium tuberculosis* complex. All these tested negative by qPCR and ddPCR, hence the primers were highly specific and exclusive to *Mycobacterium tuberculosis*. For sensitivity testing, we used already cultured *Mycobacterium bovis* BCG, *Mtb.* H37Ra and H37Rv. All three strains tested positive by both qPCR and ddPCR assays.

### 3.2. Annealing Temperature Optimization Results

Droplet amplitudes in the duplex assay dropped when an annealing temperature between 65 °C to 55 °C was run using ddPCR Supermix for probes (no dUTP), as shown in Figure S1. This was thought to be caused by the increase in time from the normal 1 h 55 min 27 s to 2 h 39 min 3 s observed due to the temperature gradient. An annealing temperature between 61.1 °C and 58.8 °C showed better separations with less rain when compared to the other temperatures. Hence, a temperature of 60 °C was chosen for further experiments using the duplex assay.

### 3.3. Droplet Separation and Target Identification

**Figure 2:**
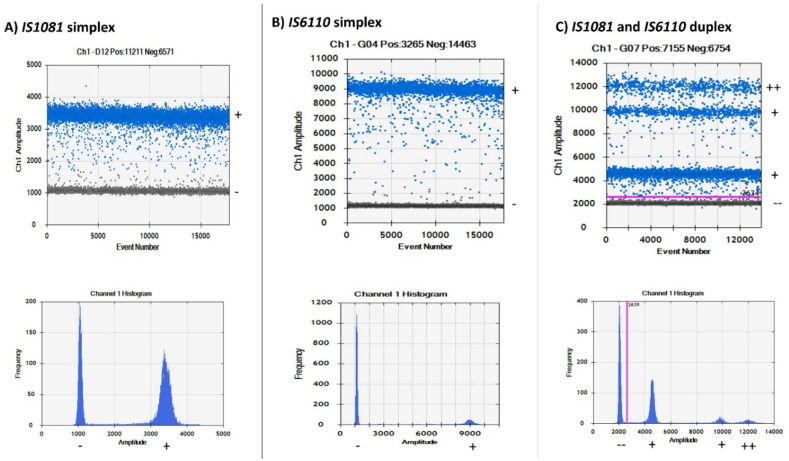
Channel 1 and histogram results for droplet separation based on simplex and duplex assays. (**A**) Droplet separation based on the *IS1081* simplex assay results. Positive droplets were observed at an amplitude of about 3500 while negative droplets were observed at an amplitude of about 1000. (**B**) Droplet separation based on the *IS6110* simplex assay results. Positive droplets were observed at an amplitude of about 9000 while negative droplets were observed at an amplitude of about 1000. (**C**) Droplet separation based on a duplex assay containing primers for both *IS6110* and *IS1081* genes. Four droplet partitions observed with double negative droplets at an amplitude of about 2000, *IS1081* droplets at an amplitude of about 4500, *IS6110* droplets at an amplitude of about 10,000, and double positive droplets at an amplitude of about 12,000. (−) negative droplets, (+) positive droplets, (++) double positive droplets, (− −) double negative droplets.

Four partitions of droplets were observed (Figure 2C) in the fluorescence amplitude when a duplex assay containing primers specific for both *IS6110* and *IS1081* genes was run. The QuantaSoft™ software could not accurately discriminate at which point the positive droplets began and to which gene they belonged. In order to discriminate and correctly identify the amplitude at which the specific genes were located, we ran a simplex assay of the two target genes separately. Consequently, two droplet partitions with a clear difference in fluorescence amplitude were observed in both the two simplex assays (Figure 2A,B). The QuantaSoft™ software could correctly discriminate positive droplets from negative droplets when the simplex assay was performed. Further analysis of the simplex assays showed a lower amplitude of positive droplets (amplitude of about 3500) in the *IS1081* simplex assay and a higher amplitude of droplets (amplitude of about 9000) in the *IS6110* simplex assay, as shown in Figure 2A,B, respectively. When the simplex assays were compared to the duplex assay using the thresholding tool, it was clear to see that the lowest droplets (amplitude of about 2000) were the double negative droplets partition, the second lowest droplets (amplitude of about 4500) were the *IS1081* gene partition, the upper droplet partition (amplitude of about 10,000) above *IS1081* partition was the *IS6110* gene partition, and the highest fluorescence amplitude droplets (amplitude of about 12,000) were the double positive droplets, as highlighted in Figure 2C. 

Additional confirmation was also achieved when the distance in fluorescence amplitude was calculated for the simplex assays result and compared to the duplex assay result. The difference between the positive and negative droplets in the *IS1081* simplex assay was estimated to be approximately 2500 (3500–1000); this was the same when the difference was calculated in the duplex assay (4500–2000). The same was also observed in the *IS6110* assay that had a difference in amplitude of about 8000 in both the simplex (9000–1000) and duplex (10,000–2000) assays. These calculations further confirmed the correct position of *IS1081* and *IS6110* droplets within the duplex assay. A 2D channel compared to the 1D channel was also used to view the droplet separation, as shown in Figure S2. The 2D amplitude and 1D amplitude separation matched perfectly with the help of the thresholding tool. Comparison of the duplex ddPCR assay to the duplex qPCR assay yielded interesting results. As shown in Figure S2, the duplex ddPCR assay could distinguish the genes separately based on amplitude while the qPCR duplex assay could only generate a single curve that did not distinguish the two genes but rather merged them into a single signal.

### 3.4. Primer and Probe Concentration Tests

**Figure 3:**
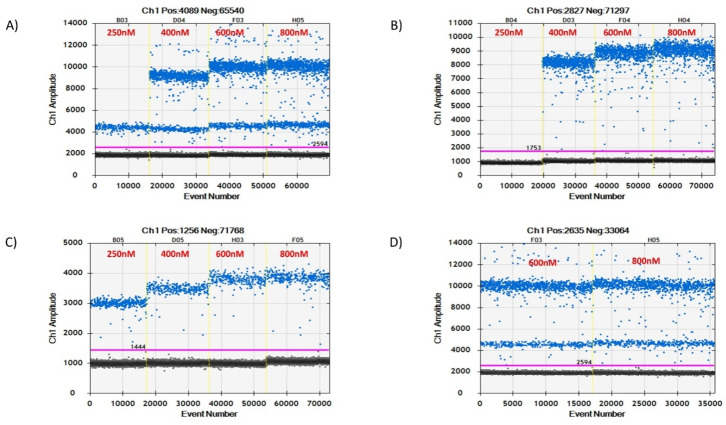
Primer concentration test of both simplex and duplex assays. (**B**,**C**) represent simplex assay results of both *IS6110* and *IS1081* genes, respectively, using various primer concentrations ranging from 250 nM to 800 nM. (**A**) is a duplex assay result containing both genes in a single reaction well. (**D**) is the optimal concentration result to be used in subsequent experiments.

To confirm the best primer and probe concentrations for the duplex ddPCR experiment, simplex and duplex assays were performed using varying concentrations of primers (250 nM to 800 nM). For primer concentration results, the overall fluorescence amplitude of positive droplets increased with an increase in primer concentrations, as shown in Figure 3. At low primer concentrations (≤250 nM), we could only detect the *IS1081* gene droplets in both the simplex and duplex assays. No droplets were detected for the *IS6110* gene at 250 nM concentrations, as seen in Figure 3A (duplex) and Figure 3B (simplex). The *IS6110* gene could, however, be detected in both duplex and simplex assays when the primer concentrations were adjusted to ≥400 nM concentrations. For the *IS1081* gene, positive droplets were observed across all concentrations, including 250 nM concentrations as shown in Figure 3A,C. Primer concentrations between 600–800 nM were chosen for subsequent experiments as they showed clear and higher separation of droplets in both simplex and duplex assays as shown in Figure 3A–D.

**Figure 4:**
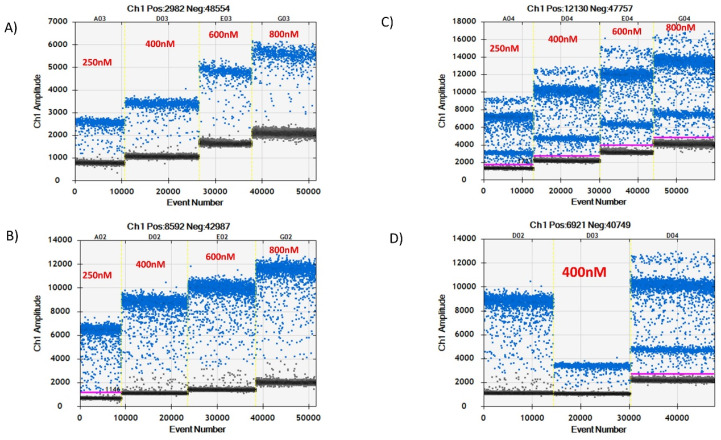
Simplex and duplex assay probe concentration (250 nM, 400 nM, 600 nM, and 800 nM) test results. (**A**,**B**) represent simplex assays results of *IS1081* and *IS6110* genes, respectively, at different probe concentrations ranging from 250 nM to 800 nM. (**C**) represents duplex assay results. (**D**) shows the result of the optimal concentration chosen for further experiments.

For the probe concentration results, an increase in probe concentrations from 250 nM to 800 nM resulted in an increase of the negative droplet amplitude, which further increased the amplitude of positive droplets as observed in both simplex and duplex assays in Figure 4. A probe concentration of about 400 nM was found optimum for further experiments as the amplitude level of negative droplets were at minimal (1000–2000) in the duplex assay. At these low probe concentrations, the positive droplets for both genes could also be determined clearly as shown in Figure 4.

### 3.5. Effect of Sample Concentration in Droplet Separation 

**Figure 5:**
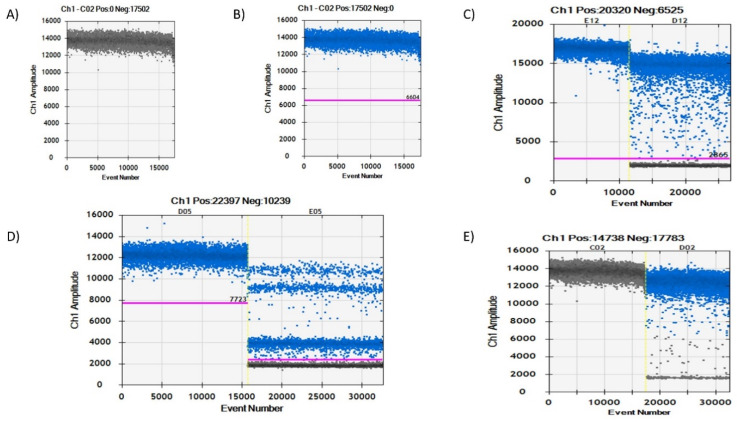
Droplet separation based on sample concentration. (**A**,**B**) represent the results of a highly concentrated sample based on automatic results (**A**) or manually generated results by thresholding (**B**) in the QuantaSoft™ software. (**C**,**E**) are the results of a simplex assay for high and low concentrated samples. (**D**) represents the duplex assay result of high (well D05) and low (well E05) concentrated samples.

High and low concentrated samples were used to determine the effect of sample concentrations in both duplex and simplex assays. For both assays, a high concentration of samples (≥60 ng/µL) resulted in a single line of droplets with an automatic result of No Call being generated by the QuantaSoft™ software. These droplets were automatically read as all negative (No Call) by the QuantaSoft™ software (black droplets in Figure 5A) or having a concentration of 1,000,000 copies/µL (blue droplets in Figure 5B) when the thresholding tool was used for analysis. Based on this, we sought to further analyze the data by diluting the samples to concentrations below 60 ng/µL. On dilution, the simplex assays produced a clear separation of the target genes from the negative droplets. This made it easy for the concentration to be determined using QuantaSoft™ software. The amplitude of positive droplets in both highly concentrated and lowly concentrated simplex assays was almost the same as observed in Figure 5C (using the thresholding tool) and Figure 5E (automatic data). This indicated that the droplets in the highly concentrated sample were not negative as read by the software automatically, but rather positive with high concentrations. The duplex assay, however, yielded interesting results when diluted. Based on the duplex single dye assay results, the highly concentrated sample was at par with the double positive droplets of the less concentrated sample (Figure 5D).

From these results, we could clearly show that the highly concentrated duplex assay droplets did not belong to either the two genes, i.e., *IS6110* and *IS1081*, but rather a mixture of the two genes per droplet that belonged to the double positive droplets amplitude, as shown in Figure 5D. The resultant diluted duplex assay also showed a clear separation of the two genes, including a double positive and double negative droplet amplitude. 

### 3.6. IS6110 and IS1081 Gene Copies

Since the copies of these genes in *M. bovis* BCG and *Mtb.* H37Ra were documented, we tried to analyze the results to confirm if one can clearly distinguish the two bacteria based on their concentration and appearance on the duplex assay without necessarily relying on the simplex assay. 

**Figure 6:**
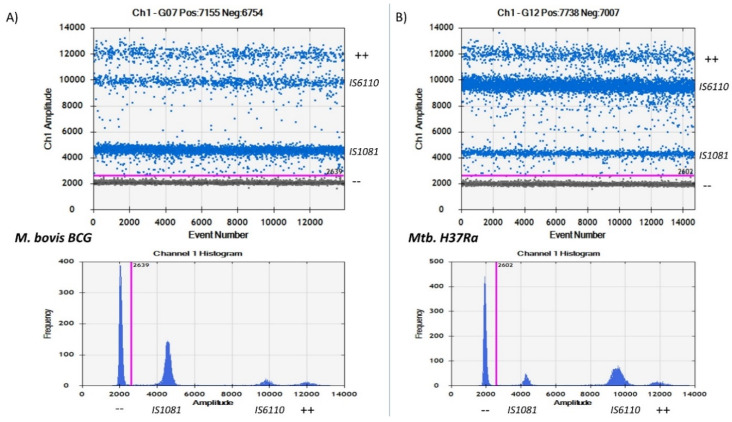
Histogram and 1D droplet concentrations of *IS6110* and *IS1081* genes based on *M. bovis* BCG and *Mtb.* H37Ra strains. (**A**) BCG result showing a high droplet concentration of *IS1081* gene than *IS6110* gene. (**B**) *Mtb.* H37Ra result showing a high concentration of *IS6110* gene droplets rather than *IS1081* gene droplets. In A, a higher concentration of droplets can be clearly observed in the *IS1081* gene amplitude in both the histogram and 1D plot when compared to double positive droplets and *IS6110* droplet concentrations. For B, the reverse is true. Less droplet concentrations can be observed in the *IS1081* and double positive droplets with high concentrations of *IS6110* droplets.

As expected, *M. bovis* BCG had more copies/µL of *IS1081* gene than *IS6110* gene, and the reverse was observed for *Mtb.* H37Ra, as shown in Figure 6. From the figure, one can also distinguish between the two bacteria without relying on the concentration values. When analyzing BCG, a high concentration of droplets can be clearly seen in the *IS1081* gene compared to the *IS6110* gene partition by using both the histogram and 1D plot. This was the reverse for *Mtb.* H37Ra, which is known to have more copies of the *IS6110* gene than the *IS1081* gene. A high concentration of droplets was clearly observed in the *IS6110* gene partition when compared to the *IS1081* partition for a duplex *Mtb.* H37Ra result. 

### 3.7. Intra/Inter-Assay Variability and Reproducibility of Simplex and Duplex Assays 

Duplex and simplex assays of *M. bovis* BCG and *Mtb.* H37Ra were run to analyze the accuracy of using the threshold tool to estimate the approximate concentration of each gene in copies/µL. Estimate values were collected by placing the threshold line above and below the genes in the duplex assay. Using the mean value and standard deviation of replicates done on both simplex and duplex assays, it was clear that the thresholding tool could accurately predict the concentration of both *IS6110* and *IS1081* genes with a low difference between their mean and standard deviation, as shown in Table 1. In both simplex and duplex assays, the difference between the actual values and estimate values of means and standard deviation was <8. For intra-assay variability, the percentage coefficient of variability (%CV) between the simplex and duplex assays was generally <6%. The inter-assay variability, however, showed high precision with a CV of <1% between the simplex assays and duplex assay results.

**Figure 7:**
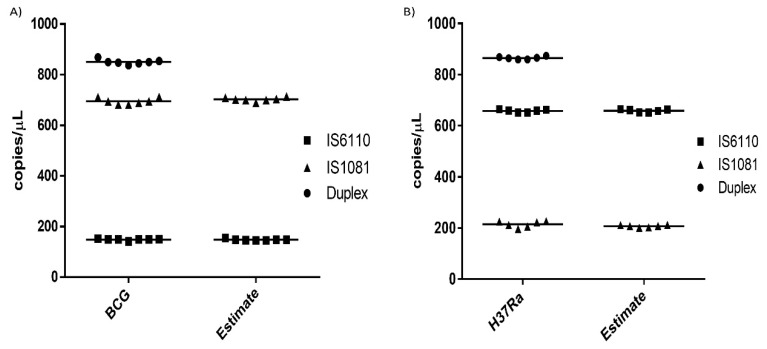
Reproducibility of replicate wells. (**A**) *M. bovis* BCG reproducibility results with BCG showing the actual duplex and simplex assay results while “Estimate” shows the approximated result calculated using the thresholding tool. (**B**) *Mtb.* H37Ra reproducibility results with H37Ra showing the actual duplex and simplex assay results while “Estimate” shows the approximated result calculated using the thresholding tool.

There was high reproducibility of both simplex and duplex assays on replicate wells, as shown in Figure 7. Estimated concentrations (using the threshold tool) and actual concentrations of both genes in different bacteria showed highly reproducible results.

### 3.8. Analysis of qPCR versus ddPCR in Detection of Samples 

Thirty samples provided by China CDC were analyzed to evaluate the ddPCR performance in the detection of real isolates. The positive predictive value (PPV) and negative predictive value (NPV) of both the qPCR and ddPCR assays were compared to culture using 7H9 medium. As seen in Table 2, both assays performed well with a 100% PPV and NPV.

## 4. Discussion

Recent reviews by Nyaruaba et al. [2] and Li et al. [4] highlight a paucity in publications on the applications of ddPCR in detecting *Mtb*. Less than 10 publications exist on the application of ddPCR towards TB detection and diagnosis [2], most of which are aimed at absolute quantification of TB using simplex and duplex double dye assays. From all these studies, however, none have highlighted the use of a single dye duplex ddPCR assay combining the two genes *IS6100* and *IS1081* in TB quantification. Furthermore, none have targeted the *IS1081* gene for the detection or quantification of tuberculosis using ddPCR. Inclusion of the *IS1081* target gene in our study was of key novel importance as the gene was used before to detect rare cases of *M. tuberculosis* strains where the *IS6110* target was found missing in clinical isolates [9,13]. This study not only aims at the inclusion of the two target genes for detecting *Mtb* but also highlights the steps toward the development and evaluation of a single dye duplex ddPCR assay that can reliably distinguish the two genes within a single sample.

The Bio-Rad QX100/QX200™ Droplet Digital™ PCR system reader was designed to detect duplex targets in two separate channels (FAM and VIC/HEX) when TaqMan hydrolysis probes are used for duplex/multiplex experiments. This means that targets for duplex/multiplex reactions were to be labeled with separate dyes and then detected in separate channels during analysis. For single channel duplexing, DNA-binding dye chemistry was used and steps toward optimization and analysis described [14]. Due to the lack of existing standards to perform, design, and analyze single dye, TaqMan-based duplex ddPCR assays, we sought to test various parameters and show the steps toward the development of such an assay suitable for detecting *Mtb* samples.

Why *Mtb.* H37Ra and *M. bovis* BCG? *Mtb.* H37Ra and *M. bovis* BCG were chosen for optimization and evaluation of the assay since these two bacteria have known copies of the target genes to be quantified. From existing literature, *Mtb.* H37Ra are shown to have 17 complete copies of the *IS6110* gene, a slight difference from the 16 copies observed in the *Mtb.* H37Rv strain [15] and 5 copies of the *IS1081* gene. *M. bovis* BCG, on the other hand, was documented to have 1 copy of *IS6110* and 5 copies of the *IS1081* gene [10]. Due to these facts, one can visually approximate the location of target droplet genes within the duplex assay, as shown in Figure 6. Having samples with known copies of the target genes to be tested in a duplex assay can help locate the genes within the duplex assay with ease. The higher the copy numbers of a particular gene, the higher the concentrations of droplets of that gene within a particular duplex assay.

**Figure 8:**
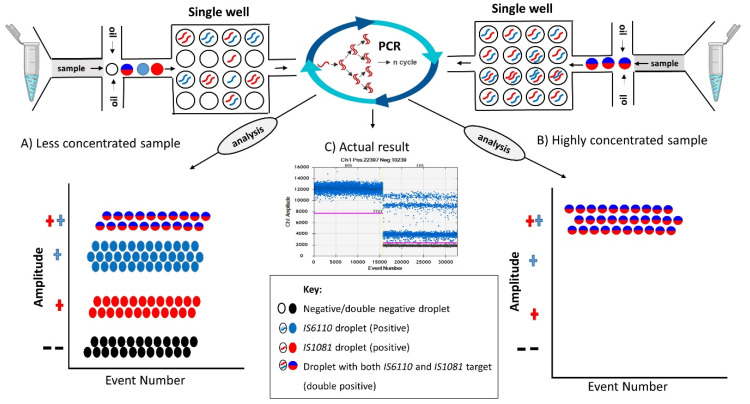
Droplet separation in a single dye duplex ddPCR assay based on sample concentration. (**A**) Less concentrated samples; droplets are generated and distributed randomly with some droplets containing only a single target (positive droplets), double targets (double positive droplets), and no targets (double negative droplets). After PCR amplification, the distribution of droplets gives four clear partitions where targets can be clearly identified. (**B**) Highly concentrated samples; each droplet generated here has both targets in a single droplet, meaning all the droplets generated are double positive droplets. PCR amplification will consequently result in highly concentrated double positive droplets that during analysis and reading will exhibit double fluorescence, which automatically results in a single double positive droplet partition.

Sample concentration plays an important role in the determination of positive, negative, double positive, and double negative droplet partitions. From our results in Figure 5, we have clearly shown that the concentration of droplets in a highly concentrated duplex single dye assay will be located in the double positive droplets and not within the individual targets as shown in simplex assays. Highly concentrated samples that were not digested/diluted will produce only single amplitudes that cannot be quantified as either positive or negative automatically by the QuantaSoft™ software. For accurate determination and quantification of droplets, dilutions of the sample to lesser concentrations will help to determine the correct position of positive droplets. Additionally, sample volume can also be adjusted after establishing the concentration of the sample. Most commercial detection assays use higher sample volumes; we believe that after the sample concentration is determined, the volume can be adjusted (e.g., 5 µL if the concentration is low or 2 µL if the concentration is high) to achieve optimum detection, quantification, and/or droplet separation results. Figure 8 is a schematic representation of how droplets separate in a duplex assay containing highly and lowly concentrated samples.

From pre-existing literature, varying primer and probe concentrations were used to distinguish target genes within a single channel using the same dye [16,17]. However, from our study, varying this concentration was shown to have very little effect on the separation of droplets within our duplex assay. This variation of results was thought to be due to differences in the efficiency of amplification for each of the targets—the lower target (*IS1081*) might have not been amplified so efficiently as the upper target (*IS6110*). Although it is a probe-based assay, the length of amplicons could also have some effect on amplitude (due to the longer time needed to amplify longer amplicons, making it less efficient in comparison to others).

The sensitivity and specificity of both the ddPCR and qPCR assays in this experiment were equal (100%). This is mostly due to the fact that the samples determined were cultured samples and not clinical isolates. We believe that this sensitivity and specificity may vary if clinical isolates are used. Especially in cases where the concentration of targets is low, more studies need to be conducted on clinical samples comparing the sensitivity and specificity of the assay to commonly used methods like sputum smear microscopy and the Xpert^®^ MTB/RIF ULTRA assay. Several studies [2], however, established the sensitivity and specificity of ddPCR in detection of *M. tuberculosis* from various clinical isolates and compared this sensitivity and specificity to other techniques like qPCR. From their findings, ddPCR was superior to qPCR in quantifying low targets from plasma [18,19,20] and sputum [3]. 

Prospectively, we think that duplex target detection and quantification using the same dye gives room for higher multiplexing and saves on the high costs incurred in simplex experiments—a technique not possible by qPCR. Using the HEX channel, one may include one or two extra targets (triplex or fourplex assay). This assay may also be used in future studies to establish if the two genes have a direct role in the pathogenesis or severity of TB infections. The assay may also be explored further in the future to test the effect of drugs on the two targets. Mass screening of a large number of patient samples may be done using this assay to also establish the epidemiology and distribution of the two targets within a population. Lastly, since our tests were mostly conducted on cultured samples; it would be of great benefit for future experiments to compare the assay’s superiority against other molecular methods like MALDI–TOF mass spectrometry.

## 5. Conclusions

Conclusively, we show steps toward the development and evaluation of a single dye duplex ddPCR assay that is capable of detecting and quantifying two targets within the same channel. The study gives room for higher multiplexing. Using the optimization steps, one may alter the target genes or organism according to their experiment. Duplexing with the two targets helps eliminate the chances of false negative results. Since no clinical isolates were used, further evaluations using real clinical isolates need to be conducted in order to explore the advantages of using a single dye duplex ddPCR assay against other methods. These studies will help improve the diagnostic toolbox regarding TB ddPCR research.

## Figures and Tables

**Table 1:** Analysis of simplex and duplex assays in replicate wells. *Mtb.* H37Ra and BCG represent the actual values read on the machine when both the simplex assay and duplex assay were run. Estimate is an approximated value calculated using the threshold tool to quantify the concentration of various genes in a duplex assay. *Mtb.* H37Ra (*Mycobacterium tuberculosis* H37Ra), BCG (*Mycobacterium bovis* BCG).

N = 6	Duplex Assay			*IS1081* Simplex			*IS6110* Simplex		
	Mean (copies/µL)	SD	%CV	Mean (copies/µL)	SD	%CV	Mean (copies/µL)	SD	%CV
***Mtb.* H37Ra**	864.500	5.431	0.628	208.5	10.910	5.240	657.5	5.468	0.832
**Estimate**	−	−	−	206.833	4.535	2.193	658	5.586	0.849
**∆ value**	−	−	−	1.667	6.375	−	0.5	0.118	−
**Inter-assay**	−	−	−	207.667	1.179	0.568	657.75	0.354	0.054
**BCG**	850.429	9.778	−	695	12.383	1.782	148.429	3.457	2.329
**Estimate**	−	−	−	702.571	7.871	1.120	147.857	3.436	3.234
**∆ value**	−	−	−	7.571	4.512	−	0.572	0.021	−
**Inter-assay**	−	−	−	698.786	5.356	0.766	148.175	0.442	0.298

**Table 2:** Comparison of ddPCR and qPCR in detection of real isolates. Positive predictive value (PPV), negative predictive value (NPV)

Method	Culture		%PPV	%NPV
	Positive	Negative		
qPCR	27	3	100	100
ddPCR	27	3	100	100

## Supplementary Materials

The following are available online at https://www.mdpi.com/2076-2607/8/5/701/s1.
