# Supplementary material for: Development and Evaluation of a Single Dye Duplex Droplet Digital PCR Assay for the Rapid Detection and Quantification of Mycobacterium tuberculosis
Source: Microorganisms. 2020 May 10;8(5):701. doi: 10.3390/microorganisms8050701 (PMC7284639; doi:10.3390/microorganisms8050701)
Supplement: Supplementary file 1 [file microorganisms-08-00701-s001.pdf]

Development and Evaluation of a Single Dye Duplex Droplet Digital PCR Assay for the Rapid Detection and Quantification of *Mycobacterium tuberculosis*

Table S1. Primer and probe sequence.

Target Gene	Primer Name	Sequence (5′-3′)	Amplicon Length	Ref
	IS6110-F	AGCGCCGCTTCGGACCACCAG		
IS6110	IS6110-R	AGGCGTCGGTGACAAAGGCCACGTA	105	[1]
	IS6110-P	FAM-CGGCTGTGGGTAGCAGACCTCACC-BHQ1		
	IS1081-F	CAGCCCGACGCCGAATCAGTTGTT		
IS1081	IS1081-R	GGTGCGGGCGGTGTCGAGGTG	102	[1]
	IS1081-P	FAM-cgcagcGGTACTCGACGCTCTGACCGACAAgctgcg-BHQ1		

F- Forward, R- Reverse, P- Probe

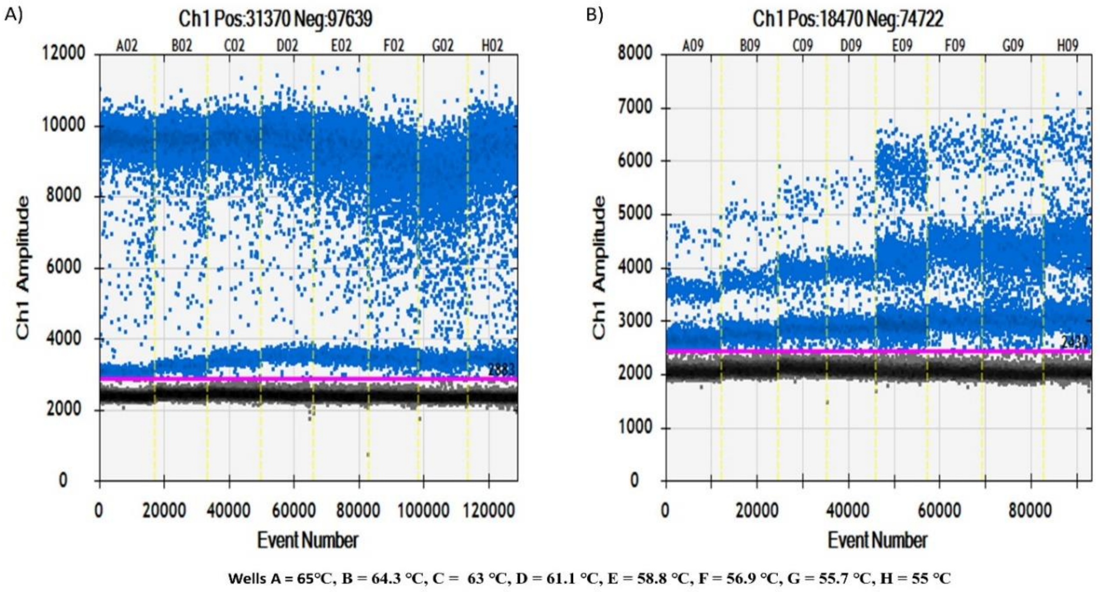

Figure S1. Temperature gradient analysis results. A) ddPCR supermix for probes results. Decrease in annealing temperature results in an increase of droplet amplitude. B) ddPCR Supermix for probes (no dUTP) results. Decrease in annealing temperature increased the positive and double positive droplet amplitudes.

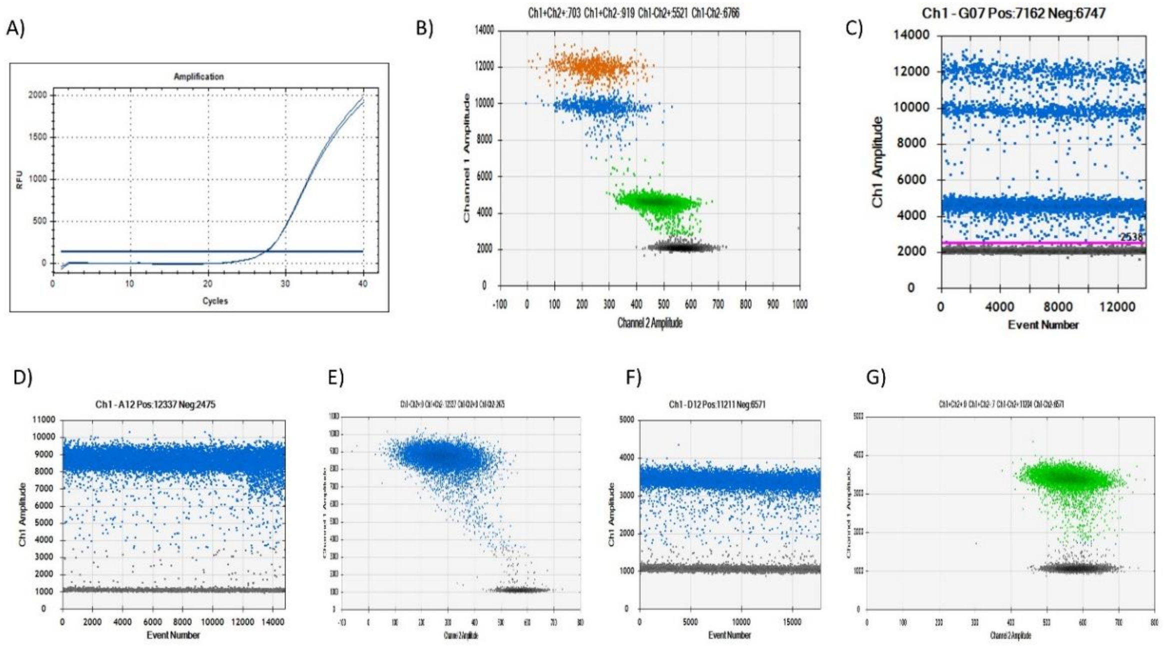

Figure S2. Channel 1 and channel 2 amplitude results compared to qPCR results. A) qPCR duplex assay result. The genes cannot be distinguished using the same dye. B) 2D amplitude showing the separation of the genes in a duplex assay. The black droplets belong to the negative droplet partition, the green droplets belong to the *IS1081* gene, the blue droplets belong to the *IS6110* gene, and the orange droplets belong to the double positive droplet partition. C) 1D amplitude of a duplex assay containing both *IS6110* and *IS10181* genes. D) *IS6110* singleplex assay 1D amplitude results. E) *IS6110* singleplex assay 2D amplitude results. Blue droplets are positive for *IS6110* gene. D) *IS1081* singleplex assay 1D amplitude results. E) *IS1081* singleplex assay 2D amplitude results. Green droplets are positive for the *IS1081* gene. NB: Diagrams C, D, and F have been explained in details in Figure 1 of the main paper.

Supplementary methods

N-acetyl-L-cysteine-sodium hydroxide (NALC-NaOH) method

Specimens spiked in sputum were briefly decontaminated and solubilized with 1~2 volumes of N-acetyl-L-cysteine (NALC) and sodium hydroxide (2% final concentration) for 15-20 minutes. Sterilized phosphate buffered saline (PBS) pH 6.8, was then added to the NALC-NaOH solution to a final volume of 50 ml before being centrifuged at 3000xg for 20 min. After centrifugation the sediment was resuspended using 2 ml PBS pH 6.8.

Temperature gradient analysis

A temperature gradient was inserted in the annealing temperature step of the PCR amplification program. A temperature gradient was run between the temperature points 55 ℃ to 65 ℃. Two supermixes were used to analyze the annealing temperature, these included the ddPCR™ Supermix for Probes and ddPCR™ Supermix for Probes (No dUTP).

Primer and probe concentration tests

Various concentrations of primers and probes were prepared to determine the effect of primers in both singleplex and duplex assays. Briefly 250 nM, 400 nM, 600 nM, and 800 nM of primers and probes were prepared and run in duplicates to determine their separation in singleplex and duplex assays. For primer concentration test. Both the two probes were kept at a low concentration of 400 nM and their respective primer concentrations varied. For probe concentration analysis, the primer

concentrations were kept constant at 800 nM and their respective probe concentrations varied between 250-800 nM.

References

1. Chakravorty, S.; Simmons, A.M.; Rowneki, M.; Parmar, H.; Cao, Y.; Ryan, J.; Banada, P.P.; Deshpande, S.; Shenai, S.; Gall, A. The new Xpert MTB/RIF Ultra: improving detection of Mycobacterium tuberculosis and resistance to rifampin in an assay suitable for point-of-care testing. *MBio* **2017**, *8*, e00812–e00817.
